# Body composition as a potential imaging biomarker for predicting the progression risk of chronic kidney disease

**DOI:** 10.1186/s13244-024-01826-1

**Published:** 2024-10-14

**Authors:** Zhouyan Liao, Guanjie Yuan, Kangwen He, Shichao Li, Mengmeng Gao, Ping Liang, Chuou Xu, Qian Chu, Min Han, Zhen Li

**Affiliations:** 1 Department of Radiology, Tongji Hospital, Tongji Medical College, Huazhong University of Science and Technology, Wuhan, China; 2 Department of Oncology, Tongji Hospital, Tongji Medical College, Huazhong University of Science and Technology, Wuhan, China; 3 Department of Nephrology, Tongji Hospital, Tongji Medical College, Huazhong University of Science and Technology, Wuhan, China

**Keywords:** Chronic kidney disease, Obesity, Progression risk, Computed tomography, Adipose tissue radiodensity

## Abstract

**Purpose:**

To investigate whether the body composition parameters can be employed as potential biomarkers for predicting the progression risk of chronic kidney disease (CKD).

**Materials and methods:**

Four hundred sixteen patients diagnosed with CKD were included in this retrospective study. Patients with a greater than 50% decline in estimated glomerular filtration rate or progression to end-stage kidney disease were in the high-risk group, otherwise, they were in a low-risk group. Body composition area, the index, and radiodensities in the Hounsfield unit (HU), which reflect the degree of X-ray absorption, were measured on abdominal CT images. Risk factors in body composition and clinical parameters of CKD were identified by Cox regression and utilized to construct the nomogram. The performance of the nomogram was assessed using time receiver operating characteristics curves, calibration curves, and decision curve analysis.

**Results:**

There were 254 patients in low-risk group and 162 in high-risk group (268 males, 148 females, mean age: 55.89 years). Urea, diabetes, 24 h-urinary protein, mean arterial pressure, and subcutaneous adipose tissue radiodensity (SATd) were valuable indicators for predicting the high-risk group. The area under curve values for the nomogram of training/validation set at 1 year, 2 years, and 3 years were 0.805/0.753, 0.784/0.783, and 0.846/0.754, respectively. For diabetic CKD patients, extra attention needs to be paid to visceral to subcutaneous fat ratio and renal sinus fat radiodensity.

**Conclusion:**

SATd was the most valuable noninvasive indicator of all body composition parameters for predicting high-risk populations with CKD. The nomogram we constructed has generalization with easily obtainable indicators, good performance, differentiation, and clinical practicability.

**Critical relevance statement:**

Radiodensity rather than an area of adipose tissue can be used as a new biomarker of prognosis for CKD patients, providing new insights into risk assessment, stratified management, and treatment for CKD patients.

**Key Points:**

- Obesity is an independent risk factor for the development and prognosis of CKD.
- Adipose tissue radiodensity is more valuable than fat area in prognosticating for kidney disease.
- Parameters that prognosticate in diabetic CKD patients are different from those in other CKD patients.

**Graphical Abstract:**

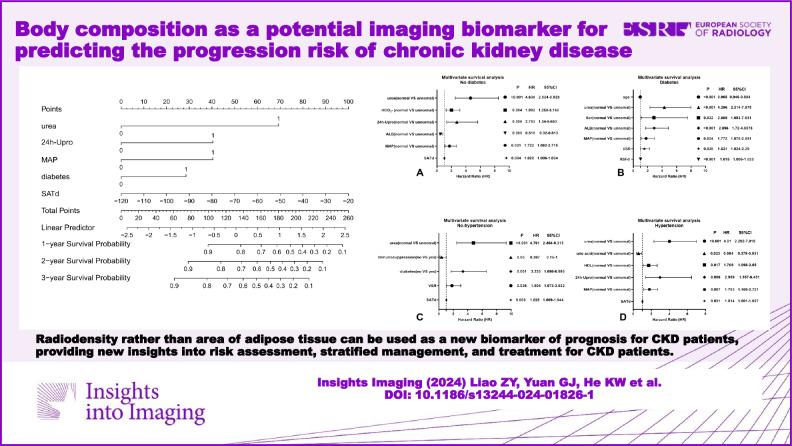

## Introduction

Global obesity rates have been rising over the past 40 years, doubling in more than one-third of countries [1]. Lancet reports that the estimated global rates of overweight and obesity are as high as about 40% [2]. As an independent risk factor for chronic kidney disease (CKD) [3, 4], obesity plays a crucial pathogenic role in 15–30% of patients [5]. In addition, the incidence rate of obesity-related nephropathy has increased from 0.2% to 2.7% in the past three decades [6]. Obesity is associated with a high cumulative prevalence of diabetes, hypertension, and cardiovascular risk which are also risk factors for CKD [7]. Therefore, obesity also needs to be urgently prevented and controlled, especially for CKD patients.

While traditional measures such as body mass index (BMI) remain the most commonly used to assess obesity, alterations in body composition are very common in CKD patients [8]. Body composition changes are the result of a combination of nutrition, energy metabolism, and inflammation which could serve as auxiliary indicators to help clinicians make better decisions for patients with CKD. Therefore, further accurate measurement and delineation of human adipose tissue is crucial. Advances in diagnostic imaging techniques have helped to determine the deleterious effects of visceral adipose tissue (VAT) [9], subcutaneous adipose tissue (SAT) [10], and renal sinus fat (RSF) [11, 12] on the onset, progression, and outcome of CKD. Visceral to subcutaneous fat ratio (VSR) provides more information about fat distribution in CKD patients than VAT or SAT alone [13]. In addition to adipose tissue, low muscle mass was also a risk factor for insulin resistance and type 2 diabetes [14]. In recent years, the radiodensity of body composition has been objectively measured by computed tomography (CT) in Hounsfield units (HU), which reflects the X-ray absorption degree of tissue and has received increasing attention as a new biomarker. For example, high adipose tissue radiodensity has been found to be related to lower survival in patients with multiple tumors [15]. However, to our knowledge, there has been no previous study focused on the relationship between the radiodensity of body composition and the prognosis of patients with CKD.

Therefore, the purpose of this study was to evaluate the progression risk of CKD by using body composition area and radiodensity measured on CT images.

## Methods

### Study subjects

**Fig. 1:**
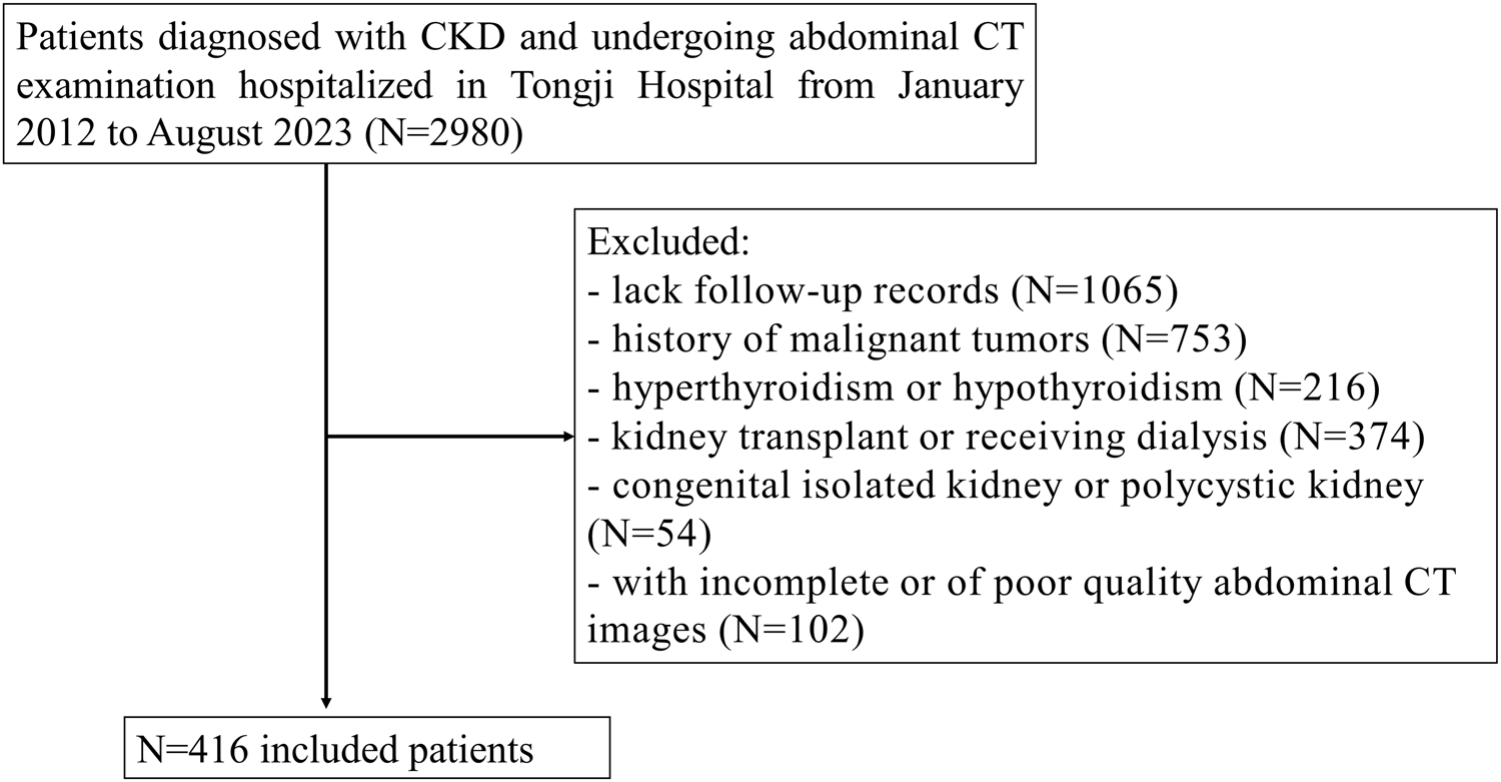
Patient selected process. CT, computed tomography; CKD, chronic kidney disease

This retrospective study was approved by the Ethics Committee of our hospital, and the requirement for informed consent was waived. This study included 2980 adult patients diagnosed with CKD and undergoing abdominal CT examination hospitalized in Tongji Hospital from January 2012 to August 2023. Exclusion criteria were as follows: (1) patients with incomplete or poor-quality abdominal CT images (serious image motion artifacts). (2) Patients with hyperthyroidism or hypothyroidism. (3) Patients with a history of malignant tumors or congenital isolated kidney or polycystic kidney. (4) Patients with kidney transplants or receiving dialysis. (5) The patient has only visited once and lacks follow-up records. Finally, 416 patients were included in this retrospective study. The patient-selected process is shown in Fig. 1. Among them, 328 patients were from the Hankou Branch (the main branch) as the training set, and 88 patients were from the Optical Valley Branch and Sino-French New City Branch as the verification set. The clinical information was collected through the medical record system of Tongji Hospital, including sex, age, height, weight, renal function index value (estimate glomerular filtration rate (eGFR), urea, serum creatinine (Scr), uric acid and HCO_3_−), four items of blood lipids (total cholesterol lipoprotein, triglyceride (TG), low-density lipoprotein cholesterol and high-density lipoprotein cholesterol (HDL)), 24 h-urinary protein (24 h-Upro), 24 h-urinary albumins (24 h-UA), albumin (ALB), fasting blood glucose (FBG), mean arterial pressure (MAP), the neutrophil-to-lymphocyte ratio (NLR), diabetes, hypertension, and use of immunosuppressive agents. BMI was calculated by dividing weight by the square of height. The follow-up endpoint event was defined as: patients with a greater than 50% decline in eGFR or progression to end-stage kidney disease (ESKD) (eGFR < 15 mL (min × 1.73 m^2^)) before the cutoff time (August 2023) were divided into a high progression risk group, others were divided into low progression risk. The overall survival time (OS) for each patient was recorded, which was, from the abdominal CT examination to the follow-up endpoint event or the cutoff time (August 2023).

### Body composition measurement

**Fig. 2:**
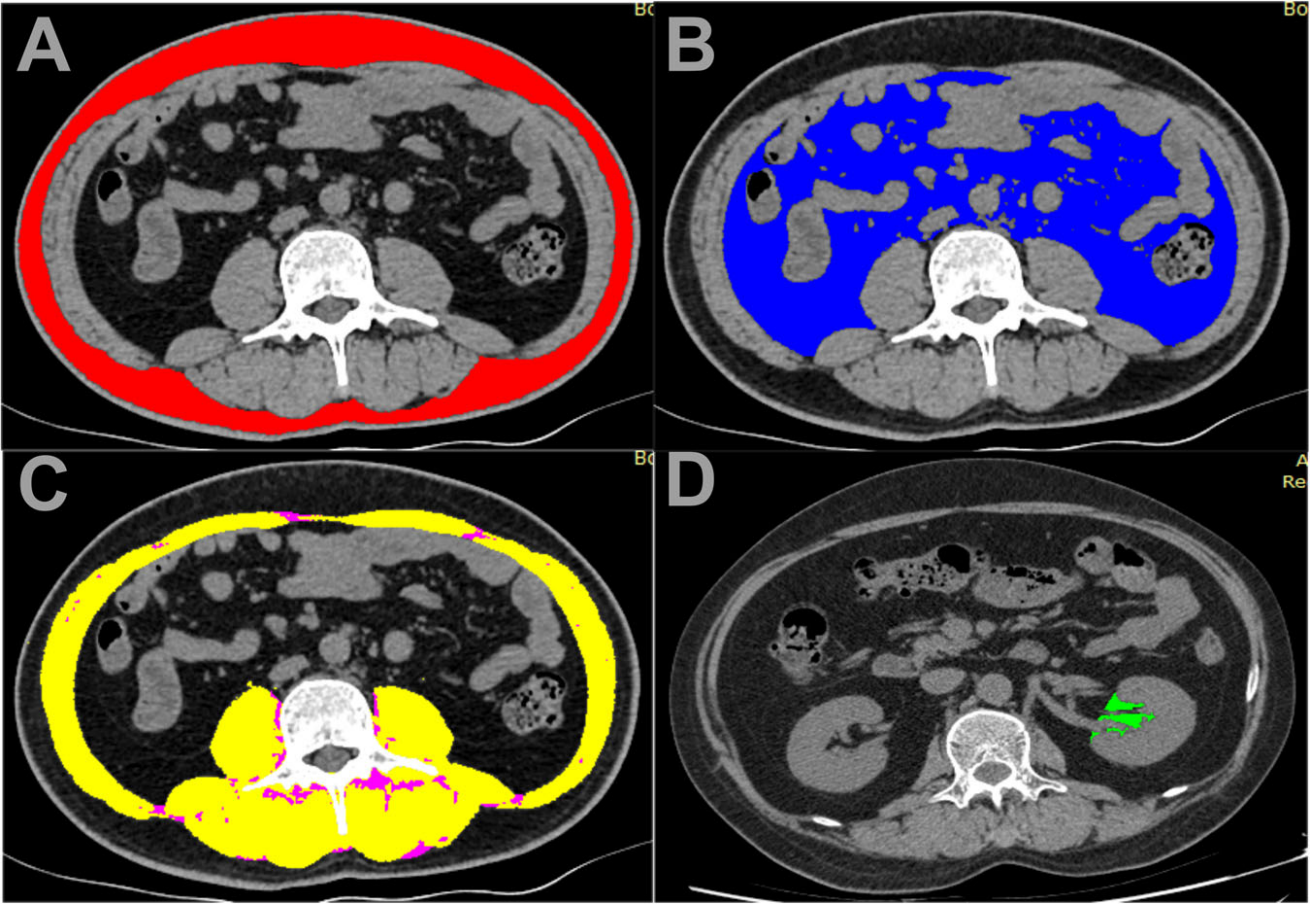
Representative cross-sectional CT images and muscle and fat of each part. **A** SAT (red); **B** VAT (blue); **C** SMA (yellow) and IIMAT (magenta); and **D** RSF (green)

All abdominal CT images were scanned by one of these CT scanners (Optima 660, GE Healthcare, US; Discovery CT750 HD, GE Healthcare, US; LightSpeed16, GE Healthcare, US; uCT 780, United Imaging, China; Aquilion One TSX-301A, Toshiba, Japan) and downloaded from the Picture Archiving and Communication System. The CT protocols: tube voltage, 120 kV; tube current, 350 mA; matrix, 512 × 512 mm; slice thickness, 5 mm; and reconstructed slice thickness, 1.25 mm. Two trained radiologists (Z.L. and G.Y.) without knowledge of the patient’s diagnosis and clinical information independently used the ImageJ software (National Institutes of Health, USA) to outline and calculate the body components. Images from 30 patients were randomly selected to assess inter-observer consistency. The middle level of the third lumbar vertebra on CT images was selected to delineate body compositions. The tissue threshold was set between −190 HU and −30 HU and then the wand tool was used to outline the edges of the VAT and SAT. Next, the boundary of VAT was manually adjusted empirically. The adipose tissue that only remains within the muscle area is intermuscular adipose tissue (IMAT). Finally, the threshold was set at −150 HU to −29 HU to outline the skeletal muscle area (SMA, including the psoas, paraspinal, and abdominal muscles). The SMA in this study was the net muscle area after the removal of IMAT. Visceral to subcutaneous adipose ratio (VSR) was calculated by dividing VAT by SAT. The fat-to-muscle ratio (FM) was calculated by the same method, that is, the sum of VAT and SAT divided by SMA. RSF of individual kidneys was measured manually in the range of renal curvature after removal of other structures at HUs of −190 to −30, using the axial CT image of the renal artery entering the renal hilum. The RSF used in this study was the mean value of RSF in both kidneys. In order to compare patients with different body types, body composition was also divided by the square of the height to derive visceral fat index (VFI), subcutaneous fat index (SFI), intermuscular fat index (IFI), skeletal muscle index (SMI), and renal sinus fat index (RFI). The mean radiodensities were collected from the same regions of interest used for body composition areas (subcutaneous adipose tissue density (SATd); visceral adipose tissue density (VATd); intermuscular adipose tissue (IMATd); skeletal muscle area density (SMAd), and renal sinus fat density (RSFd)). The body composition drawing is shown in Fig. 2.

### Statistical analysis

All statistical analyses were performed on SPSS (version 26.0, IBM SPSS, Chicago, IL, USA), GraphPad Prism (version 9.5.1, GraphPad Software Inc.), and R (version 4.4.1). The missing values of less than 30% are averaged to make up the data. Continuous variables were presented as mean ± standard deviation, while categorical variables were displayed in the form of numerical values and percentages. The clinical parameters except age were transformed into binary variables with the normal value as the dividing line. Considering the differences in body composition parameters between the different subgroups and the overall patients, univariate and multivariate survival analysis was carried out by using the original values of body composition parameters. Univariate Cox proportional hazard regression was used to independently analyze the influence of each variable on the prognosis of patients with CKD. Next, the variables considered to be statistically significant (*p* < 0.1) were included in the multivariate stepwise Cox proportional hazard regression model. Finally, the variables that had the most influence on the prognosis of patients with CKD were identified. The two-tailed significance level of *p* < 0.05 was applied. The 95% confidence interval (CI) and hazard ratio (HR) were calculated. In addition, a nomogram was drawn to predict patients with CKD survival according to the above results. A Time-dependent receiver operating characteristic (ROC) curve was drawn and the area under the curve (AUC) was calculated to evaluate the diagnostic performance of nomograms for differentiating the high-risk group from the low-risk group.

Considering the relatively small number of patients in the subgroup in the validation set, the training set and the verification set were merged into a whole for the subgroup analysis. To determine whether the same index is applicable to different subgroups and to gain an in-depth understanding of the most useful biomarkers in different subgroups, the same statistical approach as above was used to analyze male/female, diabetic/non-diabetic, and hypertensive/non-hypertensive CKD patients.

## Results

### Demographic and clinical characteristics

Four hundred sixteen patients (268 males and 148 females; mean age, 55.89 ± 16.96 years) who were clinically confirmed as CKD were included in this retrospective study. By August 2023, 162 patients with the follow-up endpoint event were classified as a high-risk group, and 254 were classified as a low-risk group, for an overall population HR of 38.9%. There were 85 cases of nephrotic syndrome, 73 cases of diabetic nephropathy, 36 cases of immunoglobulin A nephropathy, 27 cases of Henoch-Schonlein purpura nephritis, 18 cases of hypertensive nephropathy, 35 cases of membranous nephropathy, 1 case of antineutrophil cytoplasmic antibody-associated glomerulonephritis, 2 cases of obstructive nephropathy, 3 cases of ischemic nephropathy, 3 cases of interstitial glomerulonephritis, 4 cases of gouty nephropathy, 5 cases of focal proliferative glomerulonephritis, 3 cases of mild glomerulonephritis, and 121 cases of unknown causes. Among them, 88 patients in CKD stage 1, 99 patients in CKD stage 2, 113 patients in CKD stage 3, and 116 patients in CKD stage 4. The average OS of all patients was 763.01 ± 2256.02 days. The clinic information and body composition parameters of the 416 included patients are shown in Tables 1 and  2.

**Table 1:** Baseline characteristics of the population

Variables	All	Training set	Verification set
	*N* = 416	*N* = 328	*N* = 88
Sex			
Male	268	217	51
Female	148	111	37
Age, (year)	55.89 ± 16.96	55.29 ± 17.23	58.13 ± 15.81
BMI, (kg/m^2^)	23.73 ± 3.63	23.76 ± 3.64	23.69 ± 3.63
OS, (day)	763.01 ± 2260.02	811.09 ± 2530.13	629.97 ± 453.14
eGFR, (mL (min × 1.73 m^2^))	54.63 ± 35.36	59.70 ± 35.17	46.67 ± 34.28
Urea, (mmol/L)	11.22 ± 9.97	8.07 ± 5.17	16.16 ± 13.19
Scr, (μmol/L)	191.63 ± 191.69	110.15 ± 59.55	319.39 ± 249.48
Uric acid, (μmol/L)	394.14 ± 122.71	382.76 ± 119.47	411.98 ± 125.94
HCO_3_−, (mmol/L)	23.34 ± 5.41	24.01 ± 5.50	22.29 ± 5.11
TC, (mmol/L)	5.10 ± 3.47	5.51 ± 4.47	4.46 ± 2.00
TG, (mmol/L)	2.30 ± 1.97	2.34 ± 2.16	2.23 ± 1.61
LDL-C, (mmol/L)	3.76 ± 14.90	4.41 ± 18.96	2.75 ± 2.38
HDL-C, (mmol/L)	1.17 ± 1.73	1.25 ± 2.18	1.05 ± 0.46
24 h-Upro (g)	4.40 ± 12.27	3.75 ± 4.73	5.44 ± 18.73
24 h-UA, (g)	2.55 ± 3.12	2.52 ± 3.35	2.62 ± 2.71
ALB, (g/L)	33.62 ± 9.10	33.52 ± 9.98	33.79 ± 7.38
FBG, (mmol/L)	7.98 ± 4.32	7.46 ± 4.15	8.80 ± 4.48
MAP, (HHmg)	100.85 ± 17.02	97.29 ± 15.79	106.41 ± 17.42
NLR	4.95 ± 5.52	4.31 ± 3.64	5.95 ± 7.48
Hypertension			
No	169	132	37
Yes	247	196	51
Diabetes			
No	264	214	50
Yes	152	124	38
Immunosuppression			
No	333	258	75
Yes	83	70	13
Low progression risk group	254	196	58
High progression risk group	162	132	30

*BMI* body mass index, *eGFR* glomerular filtration rate, *Scr* serum creatinine, *TC* total cholesterol lipoprotein, *TG* triglyceride, *LDL* low-density lipoprotein cholesterol, *HDL* high-density lipoprotein cholesterol, *24* *h-Upro* 24 h-urinary protein, *24* *h-UA* 24 h-urinary albumins, *ALB* albumin, *FBG* fasting blood glucose, *MAP* mean arterial pressure, *NLR* the neutrophil-to-lymphocyte ratio

**Table 2:** Body composition of the population

Variables	All, (*N* = 416)	Training set, (*N* = 328)	Verification set, (*N* = 88)
VAT, (cm^2^)	121.15 ± 78.80	119.68 ± 79.78	126.64 ± 75.22
SAT, (cm^2^)	122.73 ± 63.38	119.78 ± 61.23	133.75 ± 70.10
VSR	1.08 ± 1.06	1.04 ± 0.61	1.22 ± 1.97
IMAT, (cm^2^)	9.13 ± 7.01	8.80 ± 6.33	10.35 ± 9.07
SMA, (cm^2^)	129.80 ± 32.00	132.38 ± 32.31	127.64 ± 28.71
FM	1.93 ± 1.07	1.89 ± 1.05	2.11 ± 1.12
RSF, (cm^2^)	2.83 ± 4.90	2.88 ± 5.48	2.68 ± 1.42
VATd, (HU)	−86.97 ± 14.04	−86.26 ± 13.89	−89.62 ± 14.34
SATd, (HU)	−90.17 ± 15.56	−89.43 ± 15.34	−92.93 ± 16.12
IMATd, (HU)	−58.28 ± 10.57	−57.95 ± 11.48	−59.51 ± 5.97
SMAd, (HU)	36.64 ± 9.10	37.23 ± 8.90	34.71 ± 9.68
RSFd, (HU)	−64.34 ± 30.21	−63.91 ± 29.84	−65.98 ± 31.53
VFI, (cm^2^/m^2^)	43.9 ± 27.98	43.42 ± 28.45	45.80 ± 26.21
SFI, (cm^2^/m^2^)	45.14 ± 24.00	44.06 ± 23.29	49.16 ± 26.24
IFI, (cm^2^/m^2^)	3.36 ± 2.63	3.24 ± 2.40	3.82 ± 3.34
SMI, (cm^2^/m^2^)	47.66 ± 10.02	48.01 ± 10.31	46.32 ± 8.75
RFI, (cm^2^/m^2^)	1.03 ± 1.72	1.05 ± 1.91	0.97 ± 0.51

*VAT* visceral adipose tissue, *SAT* subcutaneous adipose tissue, *VSR* visceral to subcutaneous fat ratio, *IMAT* intermuscular adipose tissue, *SMA* skeletal muscle tissue, *RSF* renal sinus fat, *SATd* subcutaneous adipose tissue radiodensity, *VATd* visceral adipose tissue radiodensity, *IMATd* intermuscular adipose tissue radiodensity, *SMAd* skeletal muscle tissue radiodensity, *RSFd* renal sinus fat radiodensity, *FM* fat to muscle ratio, *VFI* visceral fat index, *SFI* subcutaneous fat index, *IFI* intermuscular fat index, *SMI* skeletal muscle index, *RFI* renal sinus fat index, *HU* Hounsfield unit

### Interobserver reliability assessment

The intraclass correlation coefficients (ICCs) and 95% CI for each body composition parameter were as follows: VAT (ICC = 0.998, 95% CI: 0.988–0.999); VATd (ICC =0.998, 95% CI: 0.995–0.999); SAT (ICC = 1.000, 95% CI: 1.000–1.000); SATd (0.997, 95% CI: 0.992–0.999); IMAT (ICC = 1.000, 95% CI: 0.999–1.000); IMAT (ICC = 1.000, 95% CI: 0.998–1.000); SMA (ICC = 0.992, 95% CI: 0.916–0.998); SMAd (ICC = 0.989, 95% CI: 0.978–0.995); RSF (ICC = 0.996, 95% CI: 0.992–0.998); and RSFd (ICC = 0.995, 95% CI: 0.991–0.999). The results showed excellent observer agreement between the two radiologists and good measurement repeatability for body composition parameters.

### Survival analyses

**Fig. 3:**
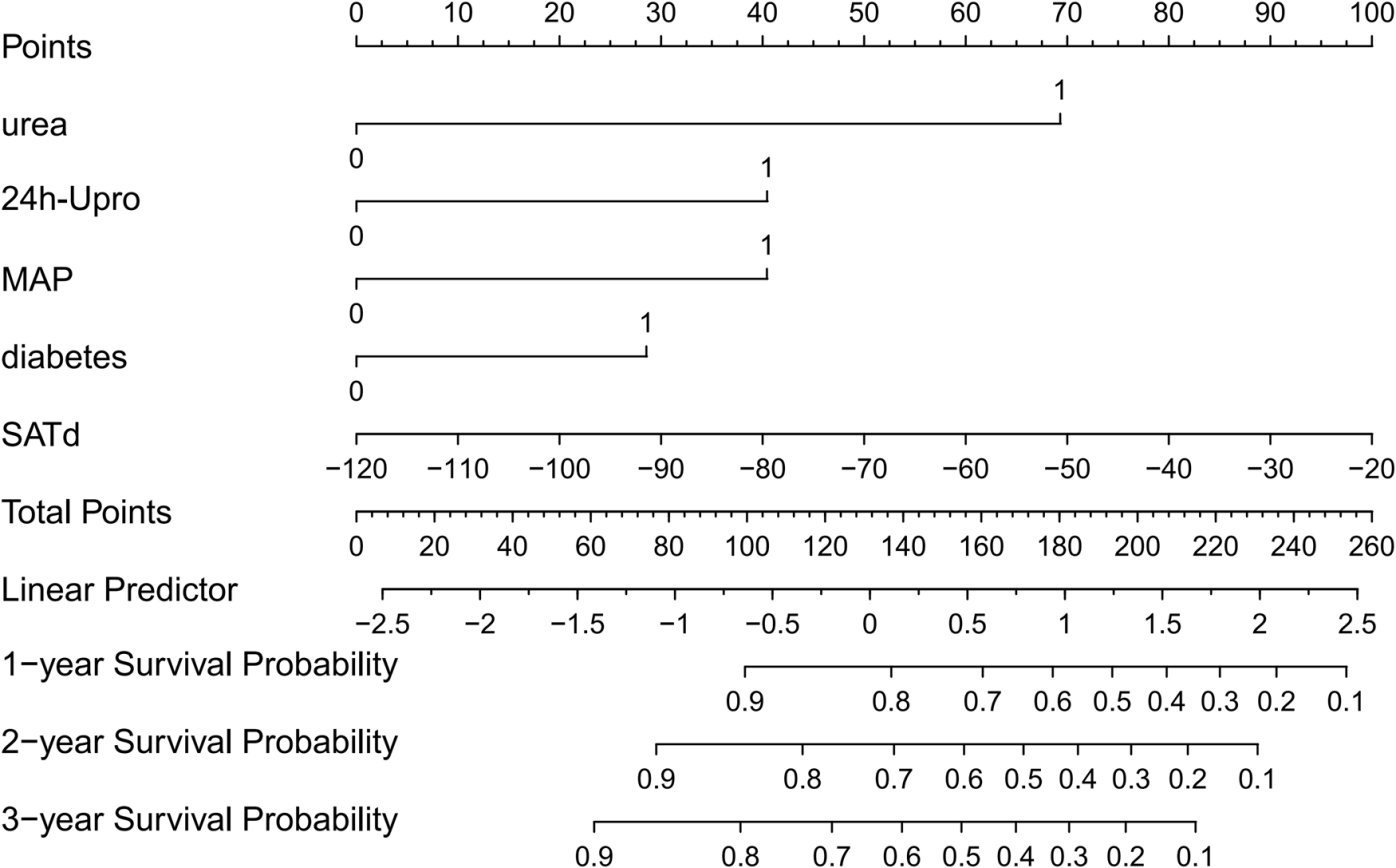
Nomogram for predicting the probability of high-risk patients with CKD. Points were assigned for urea, diabetes, MAP, 24 h-Upro, and SATd by drawing a line upward from the corresponding values to the “points” line. “Total points” are calculated as the sum of the individual score of urea, diabetes, MAP, 24 h-Upro, and SATd. MAP, mean arterial pressure; 24 h-Upro, 24 h-urinary protein; SATd, subcutaneous adipose tissue radiodensity

**Fig. 4:**
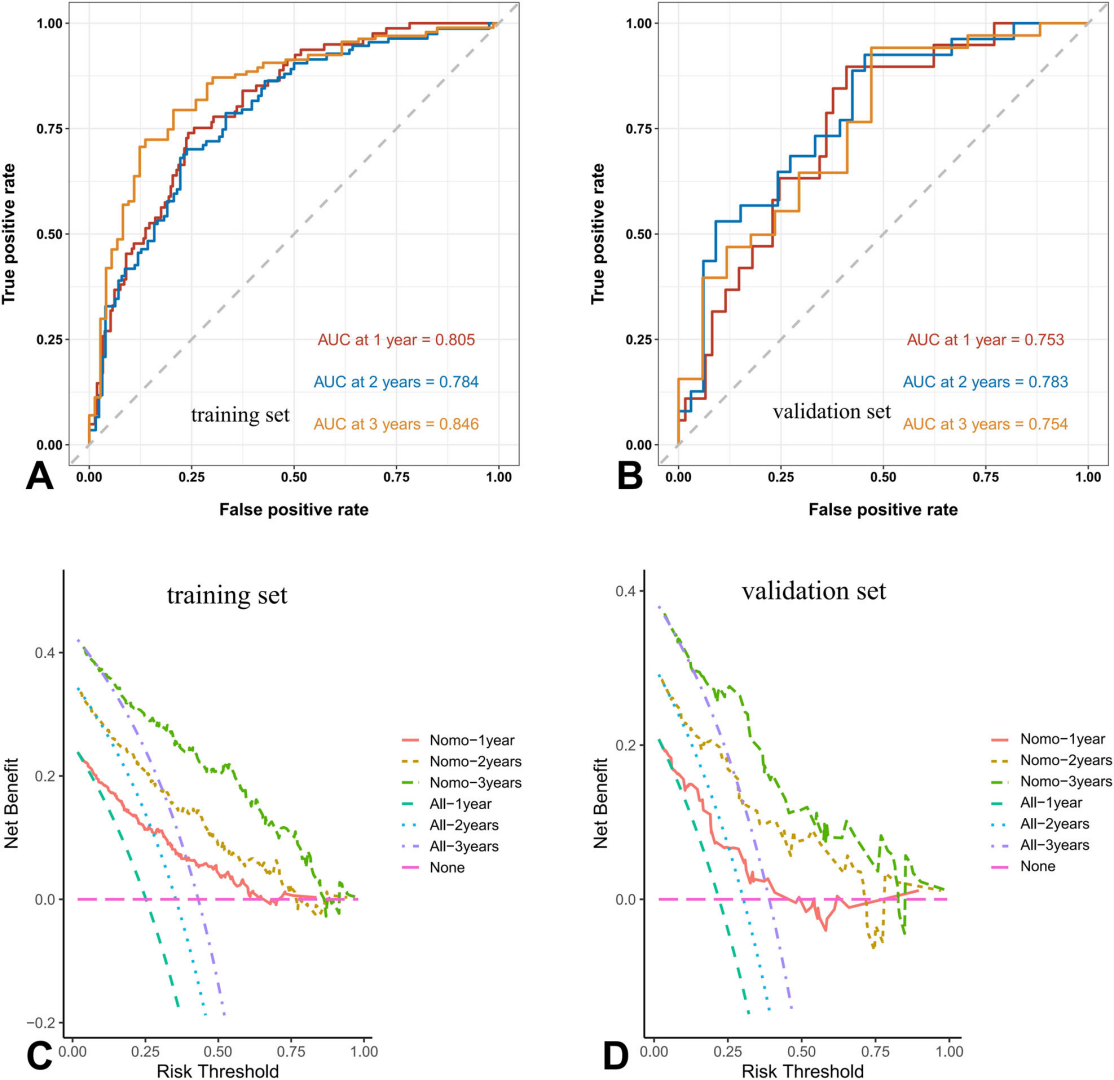
Time-ROC curves. The nomogram scores for predicting 1-year, 2-year, and 3-year high-risk groups in the training set (**A**) and validation set (**B**) were shown. The DCA of the nomogram in the training set (**C**) and validation set (**D**). The *y*-axis represented the net benefit, and the *x*-axis represented the risk thresholds for CKD patients at respective time points

**Fig. 5:**
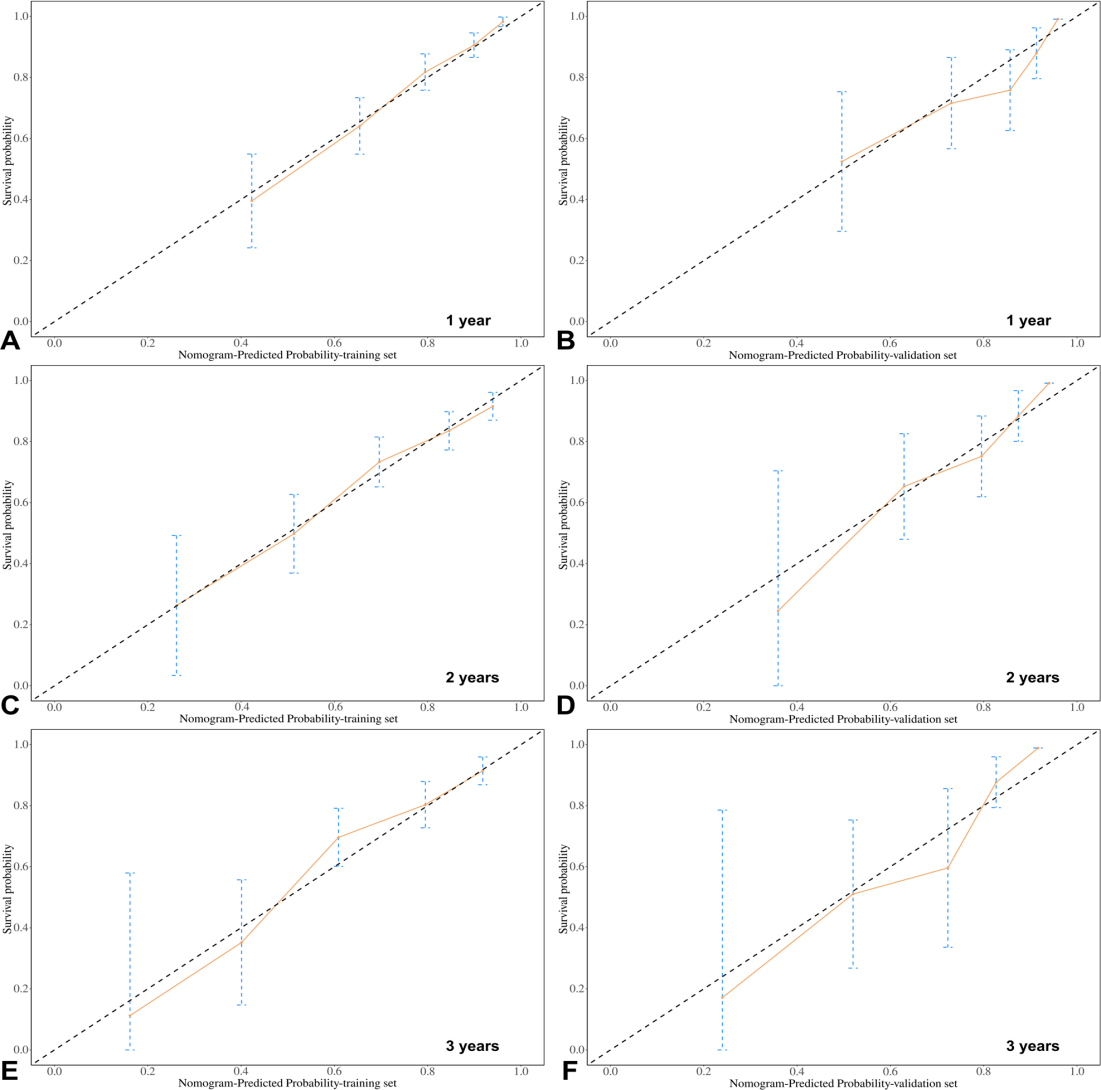
Calibration curves. The nomogram in the training set (**A**, **C**, and **E**) and validation set (**B**, **D**, and **F**) were shown. The *x*-axis represents the predicted CKD survival probabilities based on the prediction model, while the *y*-axis represents the actual outcomes from the follow-up. The diagonal gray line represented the perfect prediction of an ideal model. The solid lines represented the performance of the nomogram

For all patients, multivariate Cox analysis showed that urea (HR: 3.696, 95% CI: 2.372–5.759, *p* < 0.001), 24 h-Upro (HR: 2.253, 95% CI: 1.202–4.225, *p* = 0.011), MAP (HR: 2.112, 95% CI: 1.471–3.060, *p* < 0.001), diabetes (HR: 1.769, 95% CI: 1.246–2.512, *p* = 0.001), and SATd (HR: 1.018, 95% CI: 1.007–1.029, *p* < 0.001) were independently associated with the prognosis of patients with CKD. The survival analysis results are shown in Table 3. Figure 3 shows the nomogram constructed from the survival analysis results of the training set (Table 3). In the training set, the AUC of ROC curves for nomogram at 1 year, 2 years, and 3 years were 0.805 (95% CI: 0.751–0.857), 0.784 (95% CI: 0.722–0.844), and 0.846 (95% CI: 0.786–0.905), respectively (Fig. 4A). The verification set confirmed the good prediction performance of nomogram. The predicted AUC values for 1 year, 2 years, and 3 years were 0.753 (95% CI: 0.637–0.865), 0.783 (95% CI: 0.662–0.899), and 0.754 (95% CI: 0.604–0.900), respectively (Fig. 4B). And the clinical practicability of the nomogram was evaluated by Decision Curve Analysis (Fig. 4C, D). Figure 5 shows the calibration curve of the training and verification set (nasty 1000), which shows that the prediction results of the nomogram are closely consistent with the actual tracking results, indicating stable and extensible prediction performance.

**Table 3:** Univariate and multivariate survival analysis for predicting high progression risk patients (training set)

Variables	Univariate analysis			Multivariate analysis		
	HR	95% CI	*p*	HR	95% CI	*p*
Sex	0.836	0.579–1.207	0.340			
Age, (year)	1.004	0.994–1.014	0.460			
BMI, (kg/m^2^)	0.878	0.637–1.210	0.427			
Urea, (mmol/L)	4.466	2.881–6.923	< 0.001^*^	3.696	2.372–5.759	< 0.001^*^
Scr, (μmol/L)	4.125	2.322–7.328	< 0.001^*^			
Uric acid, (μmol/L)	1.413	0.966–2.068	0.075			
HCO_3_ −, (mmol/L)	1.867	1.317–2.645	< 0.001^*^			
TC, (mmol/L)	0.602	0.401–0.905	0.015^*^			
TG, (mmol/L)	1.050	0.742–1.487	0.783			
LDL-C, (mmol/L)	0.619	0.403–0.951	0.029^*^			
HDL-C, (mmol/L)	1.713	1.165–2.517	0.006^*^			
24 h-Upro, (g)	3.351	1.914–6.558	< 0.001^*^	2.253	1.202–4.225	0.011^*^
24 h-UA, (g)	3.468	1.869–6.434	< 0.001^*^			
ALB, (g/L)	1.149	0.844–1.565	0.377			
FBG, (mmol/L)	1.754	1.264–2.434	0.001^*^			
NLR	2.206	1.590–3.060	< 0.001^*^			
MAP, (HHmg)	1.529	1.120–2.088	0.008^*^	2.112	1.471–3.060	< 0.001^*^
Diabetes	1.836	1.298–2.596	0.001^*^	1.769	1.246–2.512	0.001^*^
Hypertension	2.018	1.381–2.949	< 0.001^*^			
Immunosuppression	0.564	0.347–0.918	0.021^*^			
VFI, (cm^2^/m^2^)	0.999	0.993–1.005	0.743			
SFI, (cm^2^/m^2^)	0.990	0.982–0.998	0.017^*^			
IFI, (cm^2^/m^2^)	0.955	0.882–1.035	0.262			
SMI, (cm^2^/m^2^)	1.015	0.999–1.032	0.071			
RFI, (cm^2^/m^2^)	1.043	0.980–1.110	0.181			
VSR	1.224	0.940–1.593	0.133			
FM	0.809	0.677–0.967	0.02^*^			
VATd, (HU)	1.015	1.003–1.028	0.011^*^			
SATd, (HU)	1.018	1.009–1.028	< 0.001^*^	1.018	1.007–1.029	0.001^*^
IMATd, (HU)	0.998	0.982–1.015	0.836			
SMAd(HU)	0.993	0.975–1.011	0.454			
RSFd, (HU)	1.000	0.995–1.006	0.932			

*BMI* body mass index, *Scr* serum creatinine, *TC* total cholesterol lipoprotein, *TG* triglyceride, *LDL* low-density lipoprotein cholesterol, *HDL* high-density lipoprotein cholesterol, *24* *h-Upro* 24 h-urinary protein, *24* *h-UA* 24 h-urinary albumin, *ALB* albumin, *FBG* fasting blood glucose, *MAP* mean arterial pressure, *NLR* the neutrophil-to-lymphocyte ratio, *VFI* visceral fat index, *SFI* subcutaneous fat index, *IFI* intermuscular fat index, *SMI* skeletal muscle index, *RFI* renal sinus fat index, *HU* Hounsfield unit, *VSR* visceral to subcutaneous fat ratio, *FM* fat to muscle ratio, *VATd* visceral adipose tissue radiodensity, *SATd* subcutaneous adipose tissue radiodensity, *IMATd* intermuscular adipose tissue radiodensity, *SMAd* skeletal muscle radiodensity, *RSFd* renal sinus fat radiodensity, *HU* Hounsfield unit

* Significant result

**Fig. 6:**
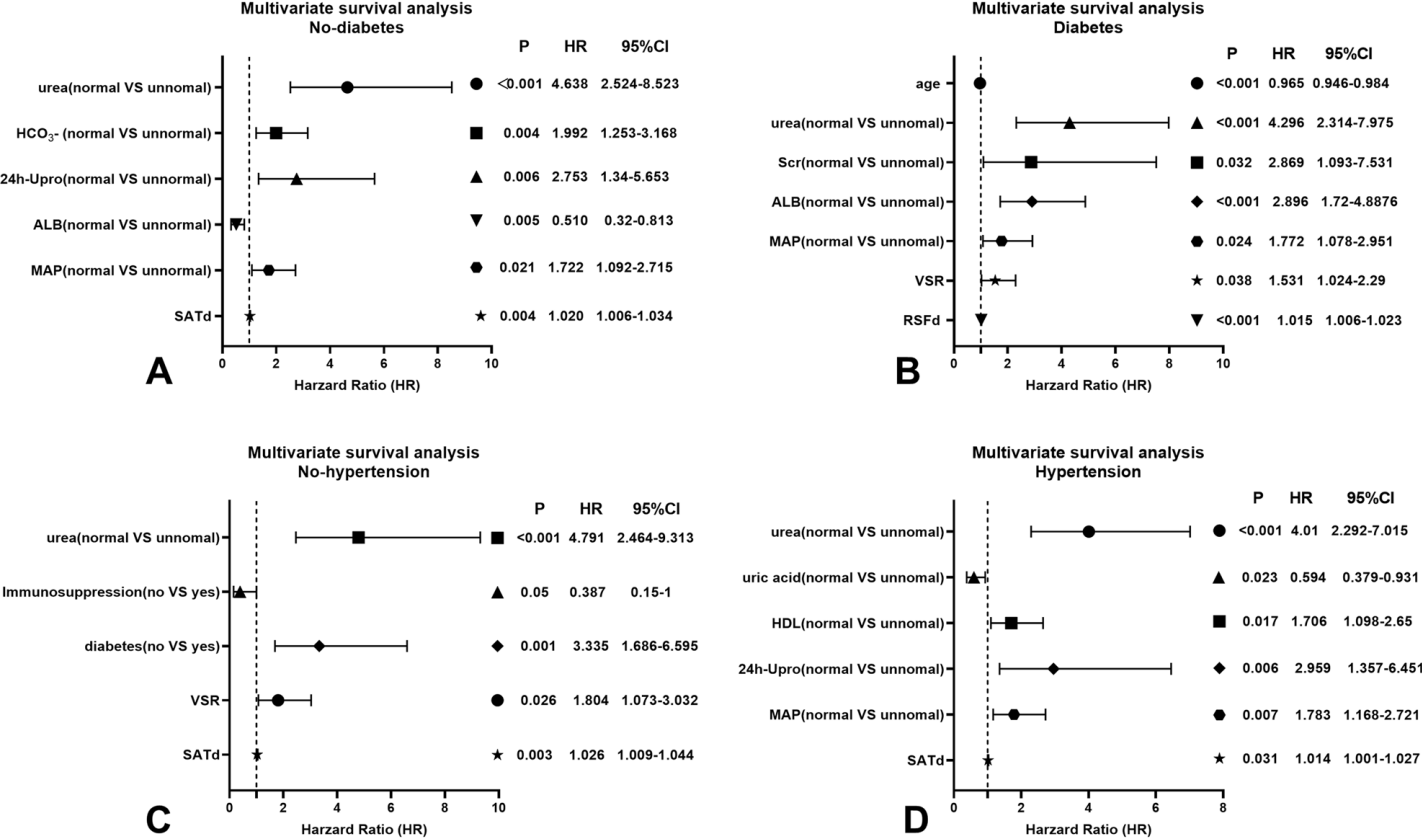
The forest plot shows the predictive ability of independent factors obtained by multivariate survival analysis in non-diabetic (**A**), diabetic (**B**), non-hypertensive (**C**), and hypertensive (**D**) subgroups. Scr, serum creatine; ALB, albumin; MAP, mean arterial pressure; 24 h-Upro, 24 h-urinary protein; HDL, high-density lipoprotein cholesterol; SATd, subcutaneous adipose tissue radiodensity; VSR, visceral to subcutaneous fat ratio; RSFd, renal sinus fat radiodensity

As shown in Table 4, urea, Scr, NLR, MAP, diabetes, and SATd were independent predictors for males with CKD, while urea, MAP, and SATd were independent risk factors for females. Figure 6 shows the independent factors affecting prognosis in diabetic/non-diabetic and hypertensive/non-hypertensive CKD patients in a forest plot. In non-diabetic patients, the multivariate model identified urea, HCO_3_−, 24 h-Upro, ALB, MAP, and SATd as significant predictors of CKD. In diabetic patients, the multivariate model identified age, urea, Scr, ALB, MAP, VSR, and RSFd as significant predictors of CKD. Urea, diabetes, immunosuppression, VSR, and SATd were independent predictors for non-hypertensive CKD patients, while urea, uric acid, HDL, 24 h-Upro, MAP, and SATd were independent risk factors for hypertensive CKD patients.

**Table 4:** Univariate and multivariate survival analysis for predicting sex-specific high progression risk patients

Variables	Males, (*N* = 268)				Females, (*N* = 148)			
	Univariate analysis	Multivariate analysis			Univariate analysis	Multivariate analysis		
	*p*	HR	95% CI	*p*	*p*	HR	95% CI	*p*
Age, (year)	0.340				0.194			
BMI, (kg/m^2^)	0.453				0.743			
Urea, (mmol/L)	< 0.001^*^	3.509	1.951–6.313	< 0.001^*^	< 0.001^*^	4.396	2.388–8.093	< 0.001^*^
Scr, (μmol/L)	< 0.001^*^	2.461	1.099–5.507	0.028^*^	< 0.001^*^			
Uric acid, (μmol/L)	0.335				0.064			
HCO_3_ −, (mmol/L)	< 0.001^*^				0.109			
TC, (mmol/L)	0.010^*^				0.825			
TG, (mmol/L)	0.600				0.881			
LDL-C, (mmol/L)	0.062				0.340			
HDL-C, (mmol/L)	0.05^*^				0.076			
24 h-Upro, (g)	0.001^*^				0.002^*^			
24 h-UA, (g)	0.001^*^				0.01^*^			
ALB, (g/L)	0.214				0.766			
FBG, (mmol/L)	0.003^*^				0.153			
NLR	0.005^*^	1.552	1.030–2.339	0.036^*^	0.433			
MAP, (HHmg)	< 0.001^*^	1.919	1.265–2.911	0.002^*^	0.001^*^	2.867	1.593–5.159	< 0.001^*^
Diabetes	< 0.001^*^	2.581	1.712–3.893	< 0.001^*^	0.132			
Hypertension	0.099				< 0.001^*^			
Immunosuppression	0.079				0.031^*^			
VFI, (cm^2^/m^2^)	0.803				0.634			
SFI, (cm^2^/m^2^)	0.234				0.070			
IFI, (cm^2^/m^2^)	0.327				0.657			
SMI, (cm^2^/m^2^)	0.187				0.184			
RFI, (cm^2^/m^2^)	0.147				0.783			
VSR	0.995				0.066			
FM	0.282				0.118			
VATd, (HU)	0.034^*^				0.087			
SATd, (HU)	0.008^*^	1.022	1.008–1.035	0.001^*^	0.001^*^	1.018	1.003–1.034	0.021^*^
IMATd, (HU)	0.623				0.857			
SMAd, (HU)	0.115				0.295			
RSFd, (HU)	0.237				0.008^*^			

*BMI* body mass index, *Scr* serum creatinine, *TC* total cholesterol lipoprotein, *TG* triglyceride, *LDL* low-density lipoprotein cholesterol, *HDL* high-density lipoprotein cholesterol, *24* *h-Upro* 24 h-urinary protein, *24* *h-UA* 24 h-urinary albumin, *ALB* albumin, *FBG* fasting blood glucose, *MAP* mean arterial pressure, *NLR* the neutrophil-to-lymphocyte ratio, *VFI* visceral fat index, *SFI* subcutaneous fat index, *IFI* intermuscular fat index, *SMI* skeletal muscle index, *RFI* renal sinus fat index, *HU* Hounsfield unit, *VSR* visceral to subcutaneous fat ratio, *FM* fat to muscle ratio, *VATd* visceral adipose tissue radiodensity, *SATd* subcutaneous adipose tissue radiodensity, *IMATd* intermuscular adipose tissue radiodensity, *SMAd* skeletal muscle radiodensity, *RSFd* renal sinus fat radiodensity, *HU* Hounsfield unit

* Significant result

## Discussion

To our knowledge, this is the first comprehensive study to explore the relationship between body composition index and radiodensity obtained from CT scans and the prognosis in patients with CKD. During our study, SATd was not only significant in multivariate survival analysis for overall patients, but also remained an independent predictor for the subgroups of male, female, non-diabetic, non-hypertensive, and hypertensive CKD patients. RSFd was meaningful for the subgroups of diabetic CKD patients, but performed poorly in the overall population. In addition, VSR was a significant predictor for non-hypertensive and diabetic CKD patients. Our results suggested that all body components need to be considered in one model and that quality rather than quantity of adipose tissue may be a new noninvasive indicator of prognosis for CKD patients. This is clinically relevant as it could help to identify high-risk patients early and stratify their management.

Radiodensity represented the quality of adipose tissue. Previous studies have shown that the radiodensity of adipose tissue measured by CT can be used to distinguish brown adipose tissue (BAT) from white adipose tissue (WAT) [16]. WAT is responsible for storing energy in the form of larger lipid droplets (also known as TGs), so WAT has larger adipose cells, higher content of simple fatty acids content, and lower radiodensity [17]. BAT is an important part of the body that produces heat [18]. The higher the radiodensity of adipose tissue, the higher the browning degree of WAT, indicating atrophy, remodeling, and injury of adipocytes. Moreover, adipose tissue with higher radiodensity contains more high molecular weight fats (including glycerophospholipids, ceramides, and other waxy fat molecules), as well as more inflammatory factors and more monocyte infiltration, which may indicate metabolic transformation in adipocytes [17]. These mechanisms ultimately affect the ability of adipose tissue to produce fatty acids and adipokines, leading to systemic metabolic dysfunction [19]. In addition, previous studies have shown that higher adipose tissue radiodensity is significantly correlated with stronger inflammatory response [20], insulin resistance, and cardiometabolic risk including hypertension [21]. All these factors contribute to deterioration and poor survival in CKD patients.

BMI had no relationship with the prognosis of CKD patients, which was contrary to the research of Garofalo et al [3]. The possible reason is that BMI, though reflective of obesity to some extent, does not distinguish between muscle and adipose tissue, let alone the distribution of adipose tissue in the body and specific adipose tissue contents [22]. Prognosis of CKD patients is associated with specific changes in the composition of one or more fat types.

Approximately 80% of the body’s fat is located subcutaneously, and subcutaneous fat mainly accumulates in the anterior abdomen, the femoral buttocks, and back [23]. Subcutaneous fat is a normal physiological buffer between the imbalance between energy intake and consumption. It mainly plays the role of storing excess fatty acids and glycerol in adipocytes in the form of TGs [24]. Compared to thigh fat, SAT in the abdominal wall absorbs more TGs and releases more free fatty acids per kilogram. The more TG stored in adipose tissue, the higher the level of leptin, which has a sustained inhibitory effect on food intake and increases energy consumption [25]. If the storage capacity of SAT reaches its limit or its storage capacity is impaired because of genetic predisposition or stress, SAT will lose its protective role [26]. The increased SATd indicated that the proportion of WAT with storage capacity decreases and the storage and protection ability of SAT is impaired. Therefore, the change of SATd was a very important biomarker for the prognosis of patients with CKD.

RSFd and VSR should be paid more attention to, for diabetic CKD patients. RSF is a kind of VAT located around the renal hilum and closely surrounding the renal vessels, renal pelvis, and lymphatic vessels. Foster et al showed that RSF has a specific role in the occurrence and development of CKD, which is independent of BMI and VAT [11]. This is because RSF can directly increase renal pressure and the various adipokines and pro-inflammatory cytokines it synthesizes and releases can act directly on renal cells through direct diffusion, resulting in renal ischemia, hypoxia, inflammation, oxidative stress, insulin resistance, and further renal fibrosis [11]. In addition, RSF can regulate arterial vascular tone and renal hemodynamics by secreting vasoconstrictor factors, similar to perivascular adipose tissue (PVAT) in skeletal muscle, thus affecting renal function [27]. One of the reasons is that increased pro-inflammatory effect or insulin resistance caused by the paracrine effect of RSF will cause a decrease in adiponectin production, which results in a decrease in smooth muscle tension relaxation [28]. Another reason is that since RSF is also a PVAT, changes in its secretory function would lead to a decrease in the production of vascular relaxation factors such as nitric oxide by endothelial cells thereby also increasing renal afferent resistance [29]. So even if the area of RSF did not increase, functional changes caused by radiodensity changes were very important for the prognosis of patients with diabetic CKD.

VSR is an important indicator of visceral obesity and is an independent risk factor for type 2 diabetes and CKD [30]. Previous studies showed that VSR is more correlated with insulin resistance and cardiovascular risk than BMI or VAT [13]. It provides more information about body composition than VAT and is the most significant value among abdominal adipose tissue, including the effect of SAT. Therefore, for CKD patients with diabetes, losing weight and controlling VSR at a low level would be an important treatment. Pioglitazone, a therapeutic drug for diabetes, could reduce the values of VSR in patients with diabetes, thereby increasing the sensitivity of tissues to insulin and glycemic control [31]. In addition, the studies of Li et al demonstrated significant differences in body composition between the sexes, as males tend to have more VAT and SMA, while females’ fat is stored primarily under the skin [32]. This shows that the normal VSR values of men and women are different in themselves. Therefore, we should pay attention to the sex of patients when evaluating the level of VSR in the clinic.

Diabetes was found to be an independent prognostic risk factor for CKD in this study. CKD Patients with diabetes were nearly two times more likely to experience adverse CKD outcomes. Previous studies have shown that diabetes has become the leading cause of CKD, accounting for approximately 45% of the causes of ESKD [33]. Hyperglycemia disrupts oxygen transport by damaging micro-vessels. In addition, up-regulation of sodium-glucose cotransporter-2 and glomerular ultrafiltration in diabetic patients leads to increased oxygen consumption due to sodium reabsorption, resulting in renal hypoxia and fibrosis [34]. Hypoxia plays a complex role in CKD, such as injury of renal tubular cells, loss of renal capillary structure through abnormal continuous activation of endothelial cells, and renal fibrosis through exfoliation and differentiation of endothelial cells into activated myofibroblasts [35]. Previous studies have shown that there is a causal relationship between the increased quality of hereditary VAT and type 2 diabetes [36], and the body composition of diabetic patients is different from that of non-diabetic patients. This may also explain why the predictive body composition of diabetic CKD patients in this study is different from that of other patients.

MAP was an independent parameter that predicts risk for prognosis in CKD patients in the overall CKD population and in subgroups of CKD patients other than those without hypertension. Hypertension is an important risk factor for the development and progression of CKD, and is common in CKD patients with a prevalence of 60% to 90%. It increases glomerular internal capsule pressure and leads to glomerular fibrosis atrophy and renal arteriosclerosis, resulting in renal parenchyma ischemia and nephron depletion, further leading to decreased renal function in patients [37]. The continued decline in renal function progresses to CKD, leading to volume overload, activation of the renin–angiotensin–aldosterone system, sympathetic hyperactivity, reduced sodium excretion salt retention, and endothelial dysfunction, which in turn leads to elevated blood pressure [37]. All of the above indicated the close relationship between CKD and hypertension and their mutual promotion. Therefore, controlling blood pressure plays a very important role in preventing and delaying the progress of CKD. Angiotensin-converting enzyme inhibitors or angiotensin receptor blockers are recommended as first-line treatment, because they are not only effective in controlling blood pressure, but also reduce urinary protein [38].

Various laboratory indicators showed good predictive ability in different classifications of patients, indicating that it is necessary to comprehensively take into account various indicators in clinical practice and prediction models, including renal function, lipids, NLR, urine protein, and ALB. They reflected the physical condition of CKD patients from different dimensions, which was consistent with the research of Liang et al [39]. Therefore, clinicians need to pay close attention to the changes in clinical indicators and control them within normal ranges, especially urea and 24 h-Upro, which were significant in all patients with CKD.

There are several limitations in this study. First, the CKD patients included were all Chinese, and it was a single-center retrospective study. Secondly, we only analyzed the effect of baseline body composition on CKD patients, which may change later as the disease progresses or is treated.

In conclusion, urea, 24 h-Upro, MAP, diabetes, and SATd were high-risk factors for poor prognosis and were independent predictors of CKD progression. The nomogram we established to predict the prognosis of CKD patients has good performance, clinical practicability, and reliability, which can provide doctors with more CKD-related biological information to guide clinical decision-making based on the existing clinical process. The prognostic value of body composition parameters varied between diabetic and non-diabetic CKD patients. The correlation and mechanism between body composition, especially adipose tissue radiodensity, and CKD need further study.

## Data Availability

The datasets used and/or analyzed during the current study are available from the corresponding author on reasonable request.

## Abbreviations

24 h-UA: 24 h-Urinary albumin
24 h-Upro: 24 h-Urinary protein
ALB: Albumin
AUC: Area under curve
BMI: Body mass index
CI: Confidence interval
CKD: Chronic kidney disease
CT: Computed tomography
eGFR: Estimate glomerular filtration rate
ESKD: End-stage kidney disease
FBG: Fasting blood glucose
FM: Fat-to-muscle ratio
HDL: High-density lipoprotein cholesterol
HR: Hazard ratio
HU: Hounsfield unit
ICC: Intraclass correlation coefficient
IFI: Intermuscular fat index
IMAT: Intermuscular adipose tissue
IMATd: Intermuscular adipose tissue radiodensity
MAP: Mean arterial pressure
NLR: Neutrophil-to-lymphocyte ratio
OS: Overall survival time
PVAT: Perivascular adipose tissue
RFI: Renal sinus fat index
ROC curve: Receiver operating characteristics curve
RSF: Renal sinus fat
RSFd: Renal sinus fat radiodensity
SAT: Subcutaneous adipose tissue
SATd: Subcutaneous adipose tissue radiodensity
Scr: Serum creatinine
SFI: Subcutaneous fat index
SMA: Skeletal muscle area
SMAd: Skeletal muscle area radiodensity
SMI: Skeletal muscle index
TG: Triglyceride
VAT: Visceral adipose tissue
VATd: Visceral adipose tissue radiodensity
VFI: Visceral fat index
VSR: Visceral to subcutaneous fat ratio
